# The analysis and optimization of thermal sensation of train drivers under occupational thermal exposure

**DOI:** 10.3389/fpubh.2023.1164817

**Published:** 2023-06-09

**Authors:** Zeyun Yang, Wenjun Zhou, Gang Xu, Xinkang Li, Mingzhi Yang, Qiang Xiao, Chaojie Fan, Yong Peng

**Affiliations:** ^1^Key Laboratory of Traffic Safety on Track of Ministry of Education, School of Traffic and Transportation Engineering, Central South University, Changsha, China; ^2^Technical Research Department, CRRC Industrial Research Institute (Qingdao) Co., Ltd., Qingdao, China

**Keywords:** train driver, thermal sensation, occupational thermal exposure, design and optimization, air supply parameters, human thermal regulation model, CFD

## Abstract

**Introduction:**

Prolonged exposure of train drivers to thermal discomfort can lead to occupational safety and health (OSH) risks, causing physical and mental injuries. Traditional method of treating human skin as a wall surface fail to observe accurate skin temperature changes or obtain human thermal comfort that adapts to the thermal environment.

**Methods:**

This study employs the Stolwijk human thermal regulation model to investigate and optimize the thermal comfort of train drivers. To minimize the time-consuming design optimization, a pointer optimization algorithm based on radial basis function (RBF) approximation was utilized to optimize the train cab ventilation system design and enhance drivers’ thermal comfort. The train driver thermal comfort model was developed using Star-CCM+ and 60 operating conditions were sampled using an Optimal Latin Hypercube Design (Opt LHD).

**Results and Discussion:**

We analyzed the effects of air supply temperature, air supply volume, air supply angle, solar radiation intensity and solar altitude angle on the local thermal sensation vote (LTSV) and overall thermal sensation vote (OTSV) of the train driver. Finally, the study obtained the optimal air supply parameters for the Heating Ventilation and Air Conditioning (HVAC) in the train cabin under extreme summer conditions, effectively improving the thermal comfort of the driver.

## 1. Introduction

The driver’s cab plays a critical role in train operation, and the driver’s thermal comfort has an important impact on ensuring the safety of train operation (1). A negative thermal comfort environment reduces driver concentration and causes mishandling (2, 3). The indoor thermal comfort problem is complex and prominent as the thermal environment of the driver’s room is influenced by a variety of factors such as temperature, humidity, and ventilation (4). Long-term exposure to thermal discomfort can affect train drivers’ occupational safety and health (OSH) (4–6). While some scholars have focused on improving the comfort of train occupants by combining physiological signals, less research has been conducted on the comfort of train drivers, with existing studies mainly simulating the flow field of the driver’s cabin without investigating the physiological response of the driver (7–9).

Batson’s research mentioned that the occupational safety and health (OSH) of heavy vehicle drivers is complicated as the transportation system is complex, while train drivers belong to heavy vehicle drivers. Moreover, as train drivers grow with the length of service, their OSH will be more severely compromised. The surrounding environment exerts an influence on the OSH of train drivers (10). With ageing societies, a certain number of older professional drivers may be employed in the future to make up for the shortage of younger workers (11). As retirement ages increase in various countries, the number of service years will increase, leading to a predicted increase in the number of ageing train drivers in the future. Occupational diseases and risks caused by exposure to fixed work environment risks over the years require urgent attention, especially in thermal environment. Improving the OSH of train drivers by enhancing the thermal comfort is an important issue that needs to be addressed as thermal environmental parameters are one of the most significant influencing factors that individuals are concerned about during the travel process. However, fewer OSH studies affected by thermal environment have been conducted on train drivers than on bus and truck drivers (11–13).

Investigating the thermal comfort of train drivers requires a suitable thermal comfort evaluation model. Zhao used the predicted mean vote (PMV) evaluation methods and the equivalent uniform temperature evaluation methods to design the air supply parameters of the air conditioner in the car, respectively (14). Sun also used the PMV model to evaluate and optimize the thermal comfort of the driver’s interior of high-speed trains (1). However, the PMV-PPD model may not provide an objective and accurate evaluation of occupant thermal comfort in highly inhomogeneous and transient temperature fields. Therefore, Zhao adopted the Berkeley model to evaluate the driver’s thermal comfort (15). The Berkeley model was developed by Zhang for predicting local and overall thermal sensations and comforts in non-uniform and transient conditions (16–18).

Scholars have mostly used Computational Fluid Dynamics (CFD) numerical simulation methods for analysis and design due to the complexity and cost of experimental methods, which are influenced by solar radiation, indoor temperature, air humidity, ventilation and air conditioning regulation (19, 20). The improvement and optimization of the HVAC system can be achieved through the design of ventilation ducts, regulation of air supply parameters, and arrangement and shape of air outlets (21). To ensure accuracy and improve optimization efficiency, scholars have also proposed combining the optimization algorithm with CFD. For example, Li introduced the adaptive network fuzzy inference system model and improved particle swarm optimization algorithm for indoor thermal comfort optimization (22). And a multi-objective optimization platform using a particle swarm optimization algorithm based on non-dominated ranking has been developed for searching multi-objective optimization results of ventilation systems in the HST cab (23). In this study, the Isight software has been applied for optimal investigation as it is mature commercial software that can optimize the thermal sensation of train drivers effectively (24, 25).

Train drivers often work on long-distance trips, which exposes them to prolonged thermal discomfort, thereby reducing their concentration and impacting their physical and mental health. Simulating the human body boundary as a fixed heat flux or constant temperature fails comprehensively consider the interaction between the human body and the surrounding thermal environment. The human thermal regulation models, such as Gagge model, Stolwijk model and Fiala model, have been developed to address this limitation (26–28). Among these models, the stolwijk has been validated and widely applied as the basic prototype of many nodal models (29).

Previous studies have shown that the OSH of train drivers is closely associated with the working environment, especially the thermal environment. The driver’s thermal sensation index accurately reflects the driver’s comfort and satisfaction with the thermal environment. To accurately evaluate the thermal sensation of the train driver and optimize the HVAC system of their room, an optimized design scheme was proposed. We performed the CFD comfort simulation of this scheme, coupled with the Stolwijk human thermal regulation model, to obtain the skin temperature of each part of the driver’s body under different operating conditions. To minimize the computational cost in the design process without sacrificing accuracy, we adopted the RBF approximation in this study (23).

The thermal comfort of train drivers is crucial for their occupational safety and health (OSH) and can affect their physical and mental wellbeing. Previous research on the thermal flow field of train driver cabins has neglected the influence of humans. Therefore, this paper proposes a more rigorous and accurate method that couples the train driver’s cabin model with the human thermal regulation model, considering the thermal physiological response of the human body. Moreover, while previous research mainly used the PMV model for stable thermal environments, this paper adopts the Berkeley model, which can calculate the comfort of train drivers under both transient and steady-state conditions. This paper also utilizes a combination of approximate model and optimization algorithm to improve the computational efficiency and effectively improve the thermal comfort of train drivers, which helps to guarantee the OSH of train drivers. The research results can be applied to the intelligent regulation of HVAC in train.

The organization of this paper is as follows: Section II describes the method and model used in this research. Section III depicts the process of modeling the train driver’s cabin with a comparison to an actual train head. Then the REF model of the train driver’s TSV with each factor is established. Paragraph IV discusses the degree of influence of environmental factors and air conditioning regulation on the driver’s TSV, and presents the optimized driver’s TSV results with the optimal air conditioning delivery parameters. Section V concludes the paper.

## 2. Method

### 2.1. Workflow of the design procedure

**Figure 1:**
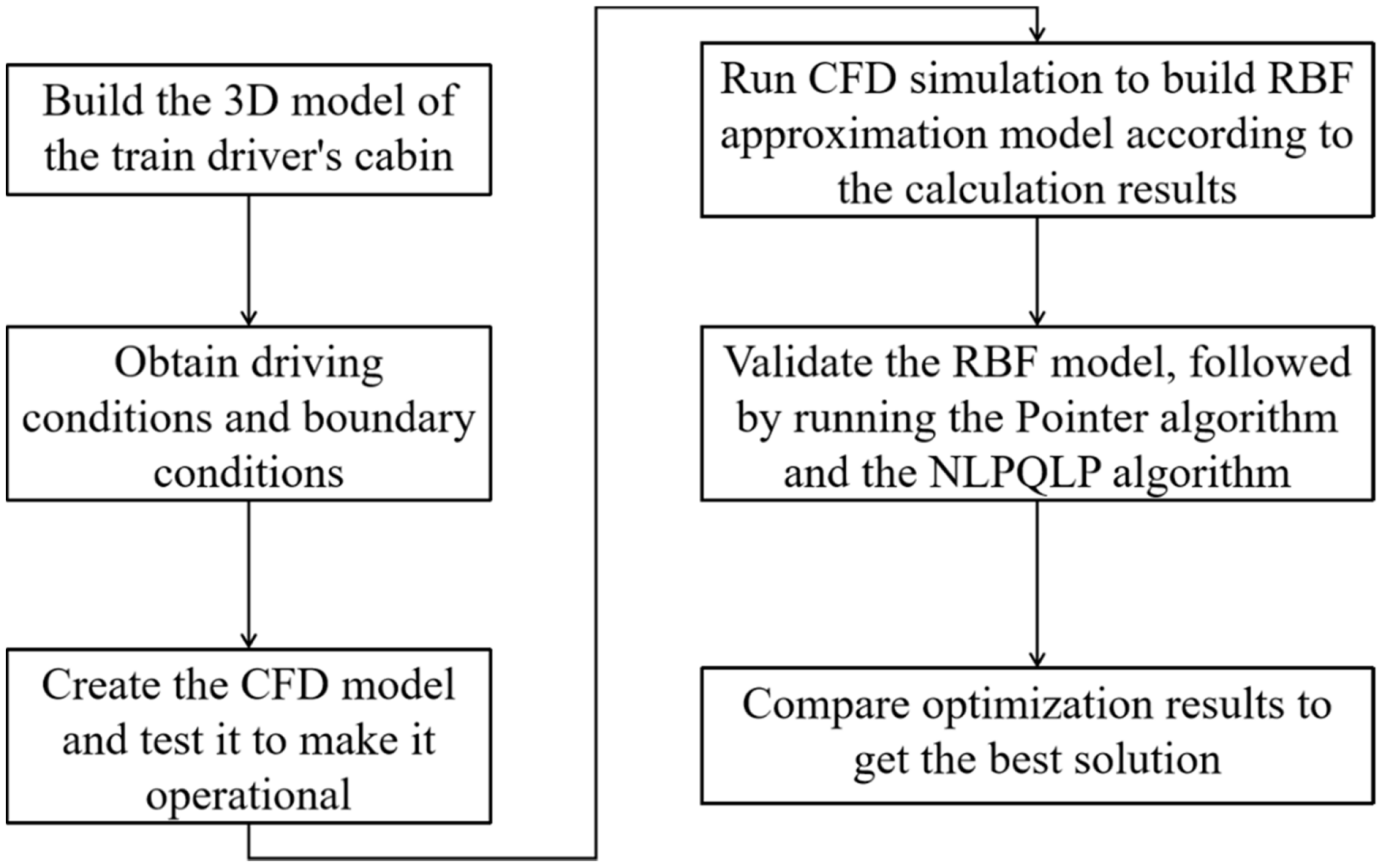
Flow chart of the research in this paper.

The research process of this paper is shown in Figure 1. First, the corresponding 3D model was established based on the actual train head size and interior arrangement. The corresponding CFD models were created based on driving conditions, thermal environment conditions, air conditioning regulations and the driver’s physiological state. The design scope and sample points were then determined using the design of experiments. The CFD simulation was run to obtain the calculation results of the sample points from which the RBF approximation model was built, followed by the validation of the RBF approximation model using the crossover method. Finally, the Pointer algorithm and the NLPQLP algorithm were compared to obtain the best optimization solution (30, 31).

### 2.2. Berkeley thermal comfort evaluation model

The overall sensation is modeled as a weighted average of the local sensation. Zhang considers that certain parts of the body should be given more weight because they are larger or more sensitive and that the sensitivity of the same body part differs on the cold and warm side. The weights also reflect local asymmetries in thermal sensation (32). The global sensation is measured on a 9-point ASHRAE scale ranging from −4 (very cold) to 4 (very hot) (33).


$$S_{0}=\frac{\Sigma w_{i}S_{i}}{\Sigma w_{i}}$$

where 𝑆_𝑖_ is the local sensation on the body part *i* obtained from people votes and 𝑤_𝑖_ is the weighting factor.


$$w_{i}=a_{i}(S_{i}-S_{m})$$

where 𝑎_𝑖_ is the parameters of the individual body parts and 𝑆_𝑚_ is the averages of the local sensations.

Zhang suggests that people’s response to asymmetric thermal environments depends on their localized thermal sensations in the body, rather than just the overall body sensation. Zhang varied the local skin temperature of each subject individually in an environmental chamber test. The local skin temperature was also measured, and both the local and overall thermal sensations and comfort levels were repeatedly assessed. Finally, the steady-state and dynamic local thermal sensation models were combined to obtain the local thermal sensation model for each body part as follows (16):


$$\begin{array}{l} Sensation_{i}=\\ \;\;\;\;\;\;\;\;\;\;4\left(\frac{2}{1+e^{-C1\left(T_{skin,i}-T_{skin,i,set}\right)-K1\left[\left(T_{skin,i}-{\bar{T}}_{skin}\right)-\left(T_{skin,i,set}-{\bar{T}}_{skin,set}\right)\right]}}-1\right)\\ \;\;\;\;\;\;\;\;\;\;+C2_{i}\frac{dT_{skin,i}}{dt}+C3_{i}\frac{dT_{core}}{dt} \end{array}$$

### 2.3. Mean skin temperature

To calculate the overall and local thermal sensation, we need a mean skin temperature (MST) calculation method. Hardy/DuBoi’s 7-sites method provides a good fit for our research as the Stolwijk model calculates local skin temperature at 14 sites on the human body. The 7-sites method was used in Zhang’s Berkeley model study (32). By combining the measurement points in this paper, we can derive the MST formula:


$$\begin{array}{l} \mathrm{MST}=0.07\times T_{\mathrm{head}}+0.35\times T_{\mathrm{chest}}+0.14\\ \;\;\;\;\;\;\;\;\;\;\;\;\;\times T_{\mathrm{left lower}\;\mathrm{arm}}+0.05\times T_{\mathrm{left hand}}+0.19\\ \;\;\;\;\;\;\;\;\;\;\;\;\;\times T_{\mathrm{left upper}\;\mathrm{leg}}+0.13\times T_{\mathrm{left lower}\;\mathrm{leg}}+0.07\times T_{\mathrm{left foot}} \end{array}$$

### 2.4. Stolwijk model of human thermal regulation

In this paper, the Stolwijk model of human physiological thermoregulation model was used. The model divides the human body into 14 segments, each consisting of a core layer (mainly composed of bones), a muscle layer, a fat layer and a skin layer. The thermophysiological model considers the effects of environmental conditions on the driver, and simulates the heat exchange processes between the human body and the thermal environment. Both passive and active systems are incorporated in the model, considering the series of physiological changes that occur in the human body in response to thermal changes (15).

### 2.5. Design of experiment

To improve the homogeneity of the stochastic Latin hypercubic design, the Opt LHD was used, resulting in a more accurate and realistic fit of the factors and responses. It enables all test points to be distributed as uniformly as possible in the design space with great space-filling and equilibrium (34).

### 2.6. Optimization algorithm

To obtain the exact optimal solution, the Pointer algorithm of Isight software was used to calculate the optimal solution. The Pointer method consists of a combination of four methods: linear simplex, sequential quadratic programming, downhill simple and genetic algorithms. The Pointer optimizer automatically captures information about the design space and automatically combines the four optimization algorithms to form an optimal optimization strategy (34).

## 3. Computational model description

### 3.1. Model preparation

**Figure 2:**
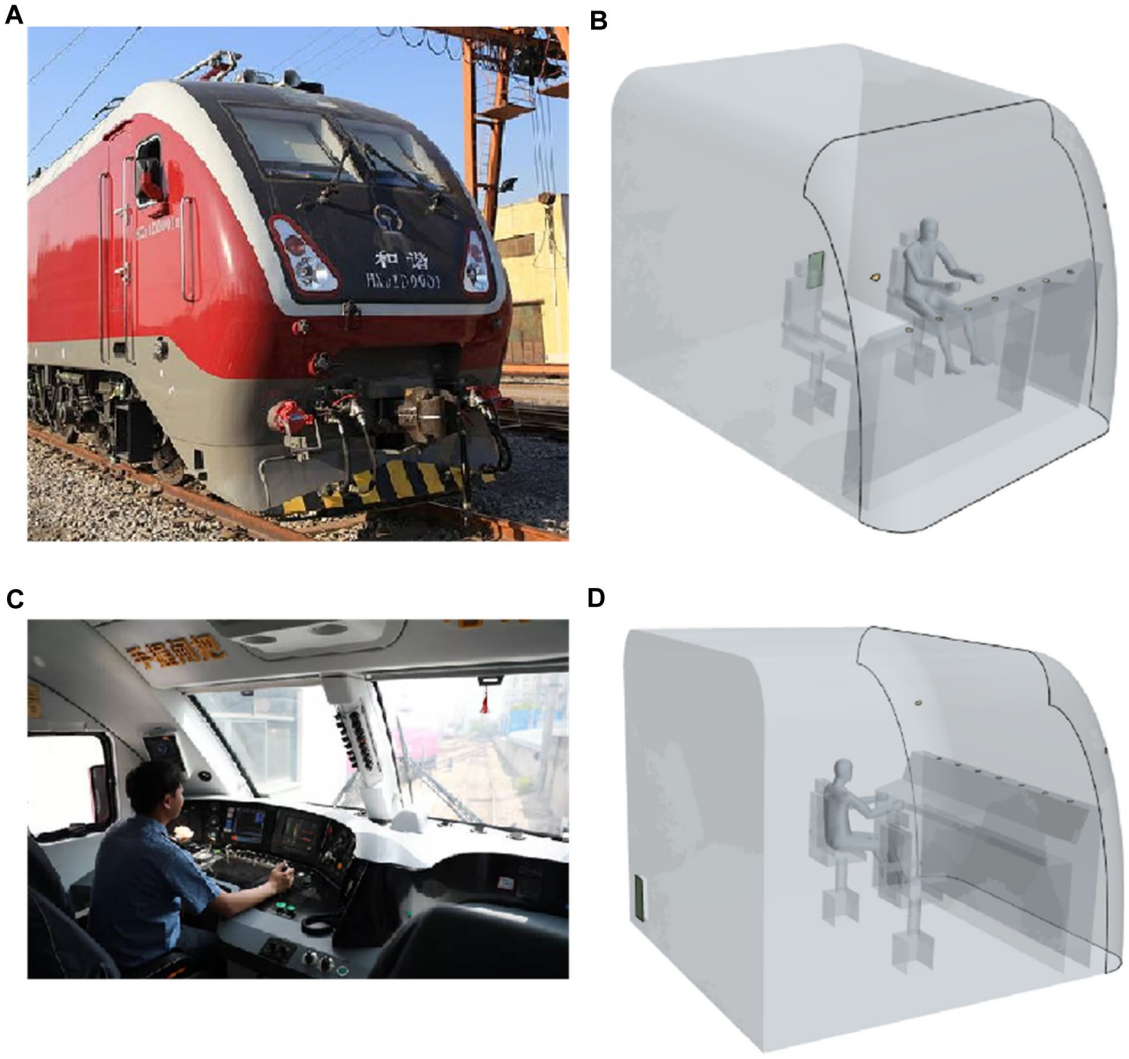
HXD1D train driver’s cab: **(A)** Head shape of the HXD1D train, **(B)** model of a train driver’s cab with driver, **(C)** interior and arrangement of real train driver’s cabin, **(D)** the interior and arrangement of the model of the train driver’s cabin.

As shown in Figure 2, the thermal comfort model of the train driver’s cabin for this study is based on the actual dimensions of a Chinese-made HXD1D train driver’s cabin, with only the enclosed area of the driver’s cabin retained and the interior simplified. There are no ducts created, but the absolute position of the driver’s seat, the operator’s console and the air vents are maintained (1). A simplified physical model of the cab is shown in Figure 2B. The air conditioning unit is installed in the engine room. There are seven air supply vents evenly arranged on the operator’s console, located between the front window glass and the control panel. As illustrated in Figures 2C,D, the actual train driver’s cabin similarly has a main driver’s seat and a co-driver’s seat, with the air vents positioned basically the same as the model. In addition, one air supply outlet is arranged on each of the left and right crossbeams at the front of the driver’s cab. Through the return air outlet on the rear wall, the air from the cab is returned to the air conditioning unit, completing the indoor air circulation (35).

### 3.2. Boundary conditions

A steady-state solution was used for the heat transfer model, an implementable K-Epsilon two-layer model was chosen for the turbulence model, the surface-to-surface (S2S) radiation model was adopted for the radiative transfer model, and the gray body thermal radiation was employed in the radiation spectrum model. The train speed was set to 120 km/h, and the thermal comfort of the train driver’s cabin was evaluated under extreme summer conditions. The heat transfer coefficient of the car body is 1.2 W/ m^2^•K and the heat transfer coefficient of the window is 1.4 W/ m^2^•K (19). The boundary conditions for the simulation calculation are shown in Table 1. The air supply volume was converted to mass flow rate as the boundary parameter. The initial temperature of the skin of each part of the driver is shown in Table 2. The height of the driver is 1.70 m, the clothing thermal resistance is 0.775 m^2^•K/W (1Clo = 0.155 m^2^•K /W), and the metabolic rate is 75.4 W/m^2^ (36).

**Table 1:** Boundary parameters for thermal comfort simulation of train drivers.

Climatic temperature/°C	Climatic relative humidity/%	Indoor relative humidity/%	Supply air temperature/°C
35	75	50	[10, 20]
Supply air volume	Supply air angle	Solar radiation	Solar altitude angle
[500,800]	π/4;π/2	[900,1,300]	[0.8,1.4]

**Table 2:** Initial temperature of each body part.

Local body part	Skin temperature (°C)	Right hand	35
Head	36	Upper Left Leg	35
Chest	35	Upper Right Leg	35
Upper Left Arm	35	Lower Left Leg	34
Upper Right Arm	35	Lower Right Leg	34
Lower Left Arm	35	Left Foot	36
Lower Right Arm	35	Right Foot	36
Left Hand	35	Right Hand	35

### 3.3. Design of experiment

**Figure 3:**
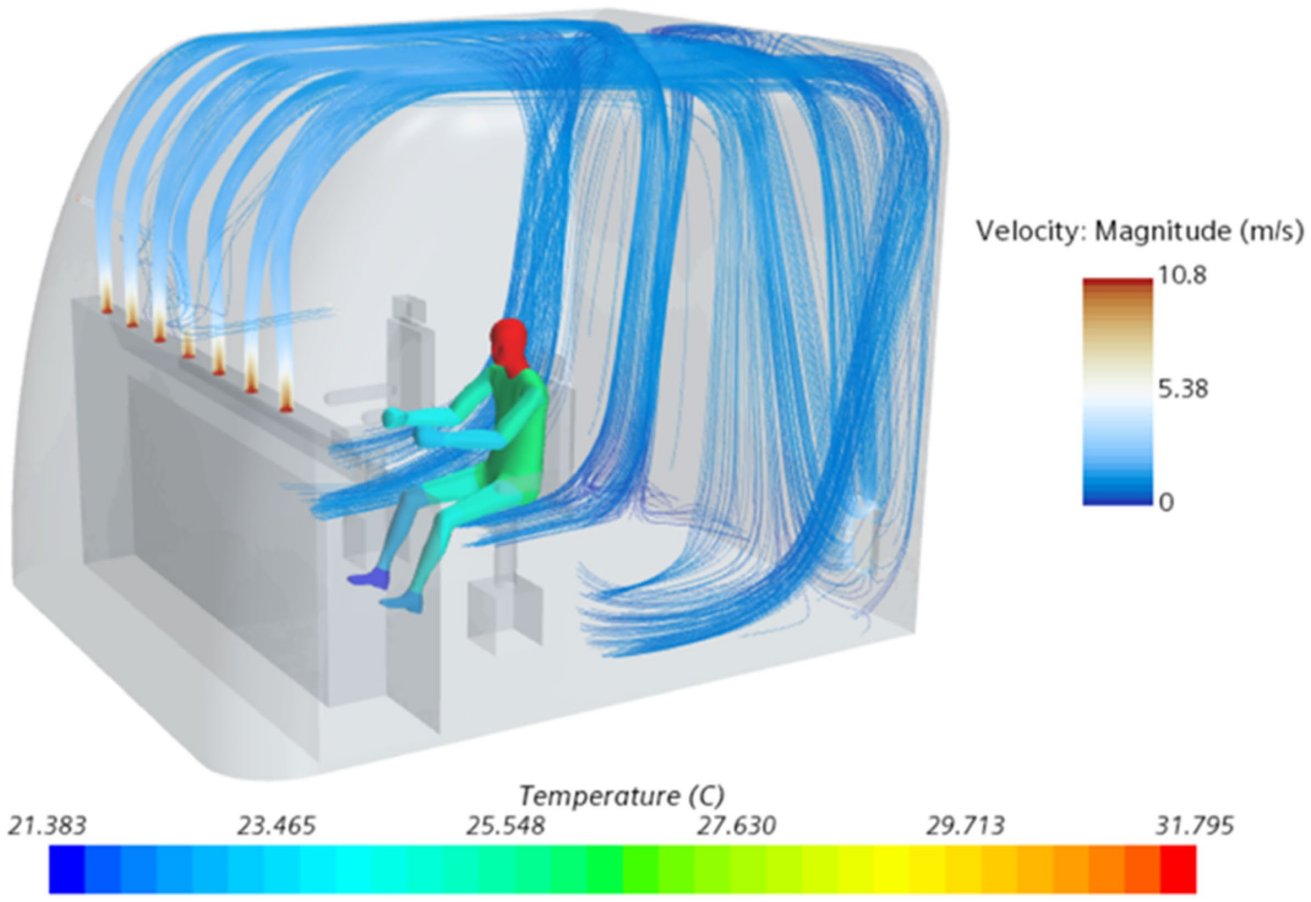
Thermal comfort calculation results for train drivers under certain working conditions.

In this section, we describe the design of experiment used to optimize the ventilation system in the driver’s cabin. We used an optimal Latin hypercube design to draw 60 sample points. To accelerate the optimization process, we employed the Radial Basis Function (RBF) approximation as a fast alternative to the time-consuming CFD simulations (37). To ensure the accuracy of the RBF prediction, we used 10% of the total number of sampling points for cross-validation error analysis (23). Figure 3 presents the calculation results of the skin temperature in different parts of the driver and the velocity of the air supply flow line under a given working condition.

**Figure 4:**
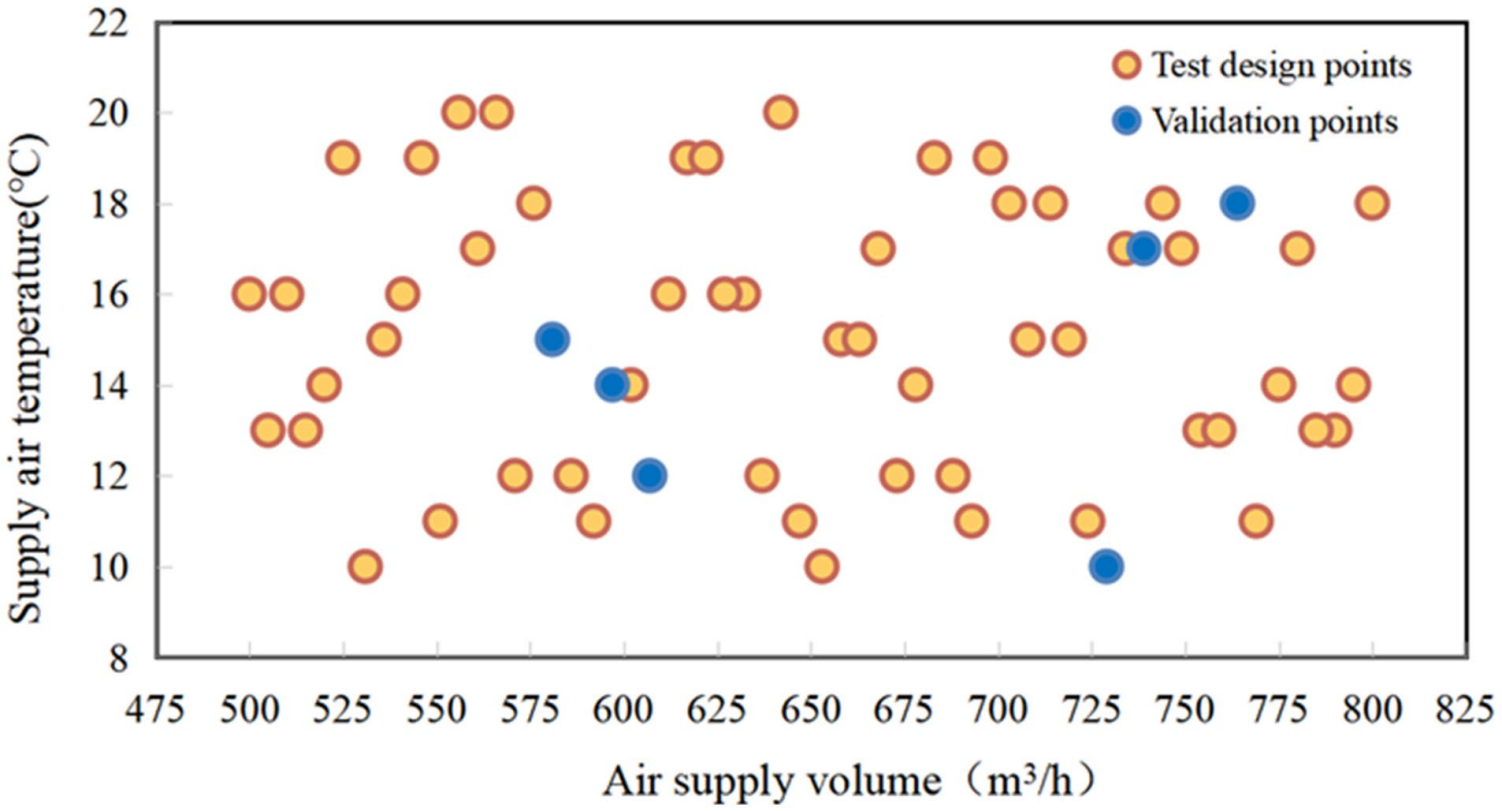
Sample points for experimental design.

**Figure 5:**
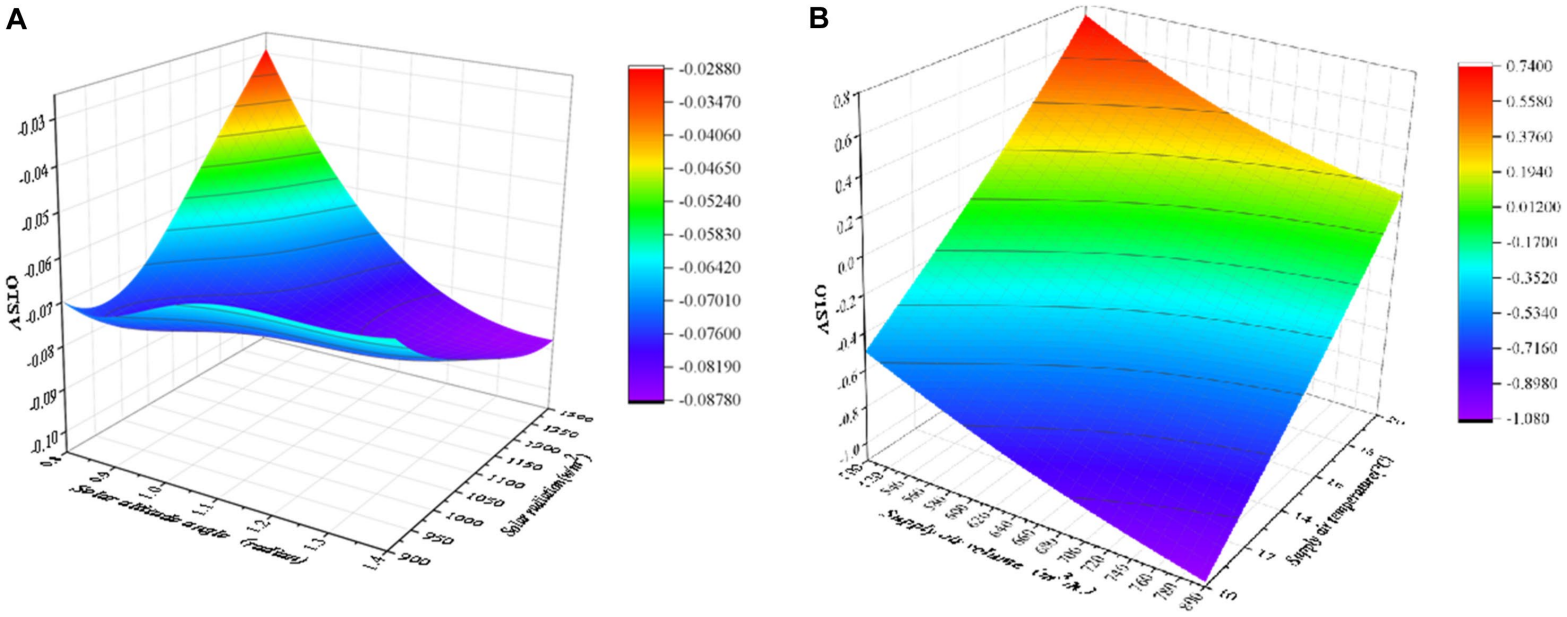
Three-dimensional diagram of the RBF approximation model of OTSV: **(A)** OTSV vs. solar radiation and solar altitude angle, **(B)** OTSV vs. air supply volume and air supply temperature.

The simulations conducted at the orange points in Figure 4 were used to train the RBF approximation. After the training, the RBF approximation method is used to predict the MST, LTSV and OTSV under the six different conditions (i.e., blue points) and validated against the CFD predictions (23). Figure 5A presents a three-dimensional graph showing the non-linear variation of OTSV with solar radiation and solar altitude angle (when the air supply angle is 90°, the air supply temperature is 16°C, and the air supply volume is about 600 m^3^/h), which reveals that the variation of OTSV is non-linear. Figure 5B shows the smoother and more regular three-dimensional graph of the variation of OTSV with air supply volume and air supply temperature (when the air supply angle is 90°, the solar altitude angle is 1 radian, and the solar radiation is 1,100 w/m^2^).

To evaluate the accuracy of the RBF model, we performed cross -validation was performed using 10% of the sample points. We used R-squared and root mean square to assess the prediction accuracy. R-squared values range between 0 and 1, where 1 indicates no error. The root mean square value should be less than 0.2. Table 3 shows the prediction error of the RBF model in comparison to the corresponding CFD results. As shown in Table 3, the R-squared values for MST, OTSV and LTSV, except for the LTSV of the header, were greater than 0.9, indicating the accuracy of the RBF approximation method in capturing the response surface of the objective value with respect to different ventilation conditions and external thermal environment conditions.

**Table 3:** The *R*-squared and root mean square of approximate model in predicting thermal sensation vote.

	*R*-squared	Root mean square		*R*-squared	Root mean square
Head	0.81	0.11	Right Hand	0.98	0.04
Chest	0.98	0.04	Left Upper Leg	0.97	0.04
Left Upper Arm	0.99	0.03	Right Upper Leg	0.98	0.04
Right Upper Arm	0.91	0.08	Left Lower Leg	0.99	0.02
Left Lower Arm	0.96	0.05	Right Lower Leg	0.98	0.04
Right Lower Arm	0.99	0.04	Left Foot	0.99	0.03
Left Hand	0.97	0.05	Right Foot	0.99	0.03
OTSV	0.996	0.02	MST	0.992	0.02

## 4. Results and discussions

### 4.1. Analysis of different air supply conditions

**Figure 6:**
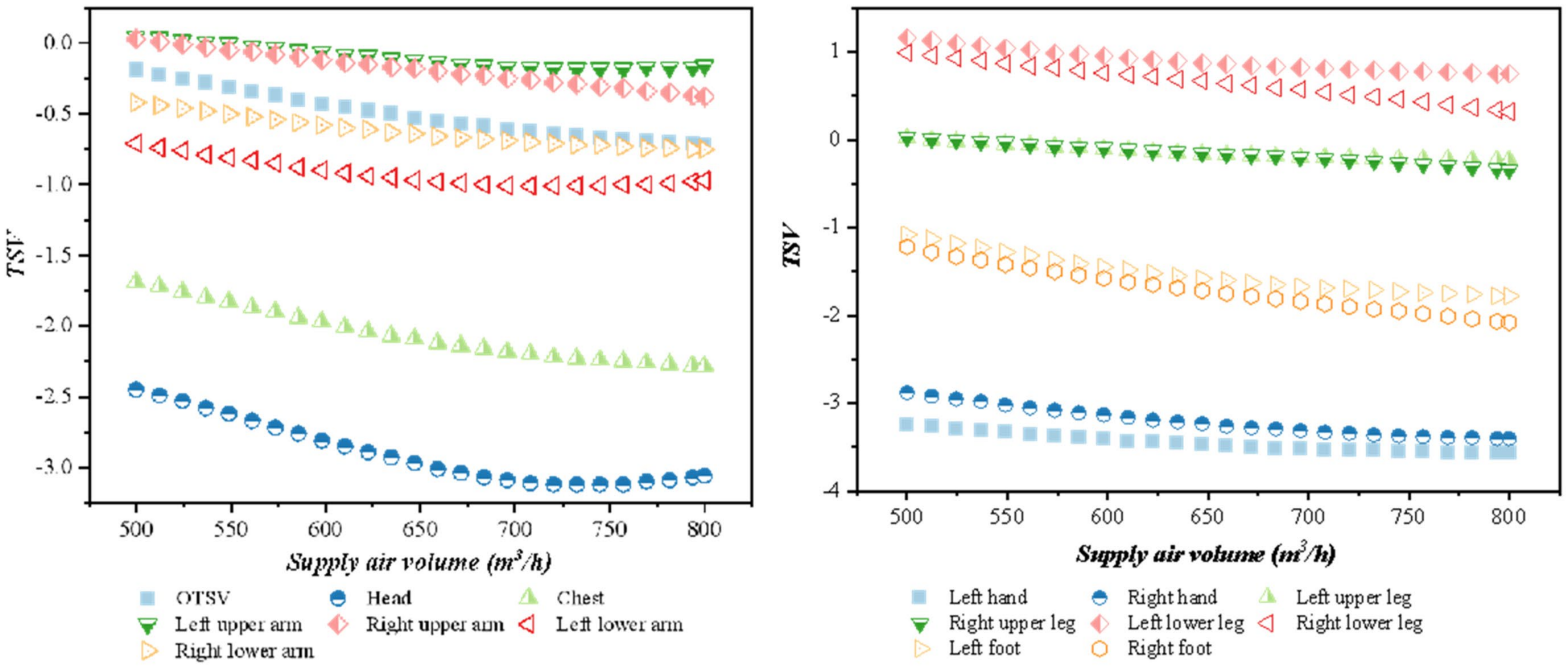
Variation of TSV with supply air volume.

The impact of air supply on overall thermal sensation and local thermal sensation was analyzed for various air supply volumes and temperatures. Figure 6 (when the air supply angle is 45°h the air supply temperature is 15°C, the solar altitude angle is 1radian, and the solar radiation is 1,100 W/m^2^) illustrates that the thermal sensation is negatively correlated with the air supply volume. Additionally, the local thermal sensation of the head and hands was lower than the other parts, while the local thermal sensation of the calves is higher. The OTSV decreases in the (0, 1) interval.

**Figure 7:**
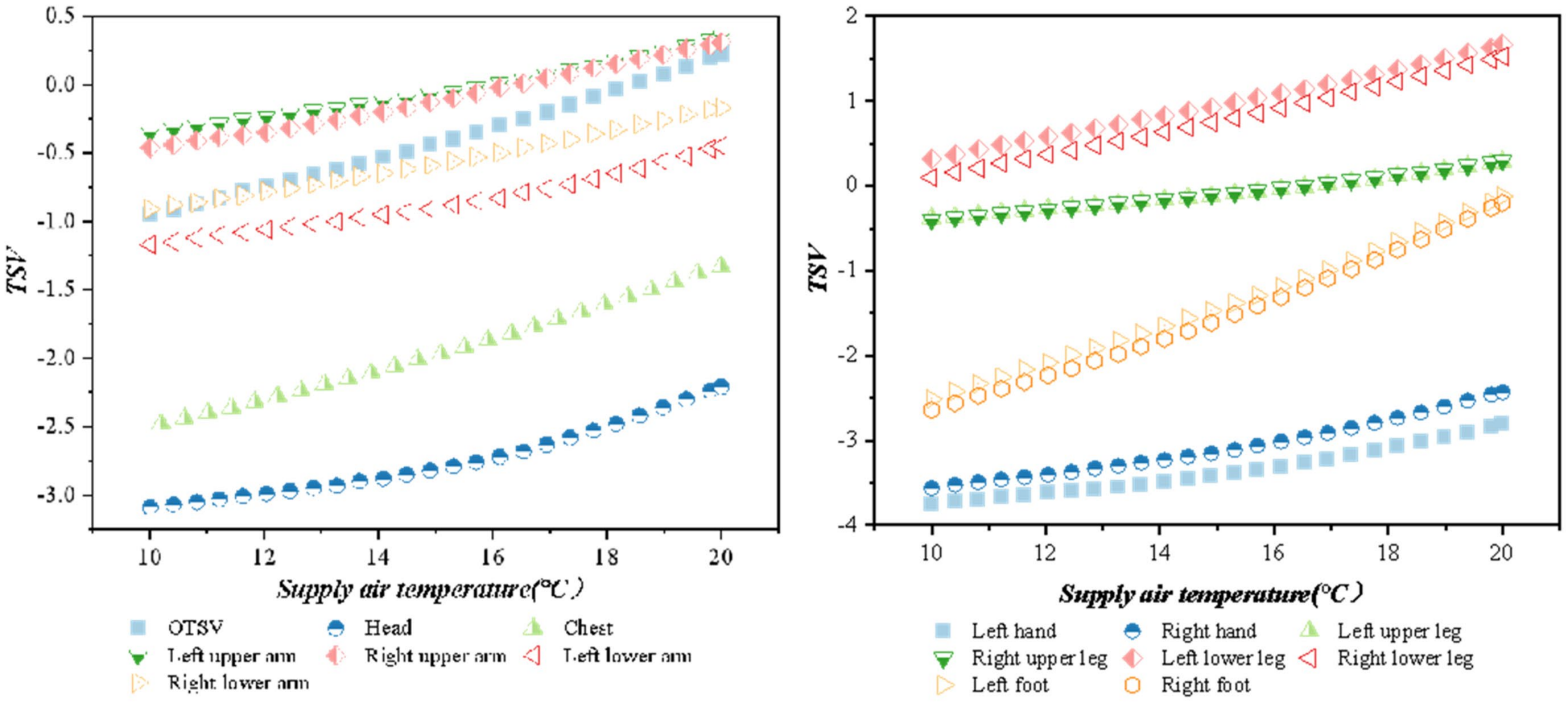
Variation of TSV with supply air temperature.

The relationship between the OTSV and LTSV with the change of air supply temperature is shown in Figure 7 (when the air supply angle is 45°, the air supply volume is 600 m^3^/h, the solar altitude angle is 1 radian, and the solar radiation is 1,100 w/m^2^) shows that thermal sensation is positively correlated with the air supply temperature, and the value of thermal sensation increases with an increase in air supply temperature. The thermal sensation of the chest and feet had the largest variation according to the OTSV increment in the (−1, 0.5) interval.

### 4.2. Solar radiation and solar altitude angle

**Figure 8:**
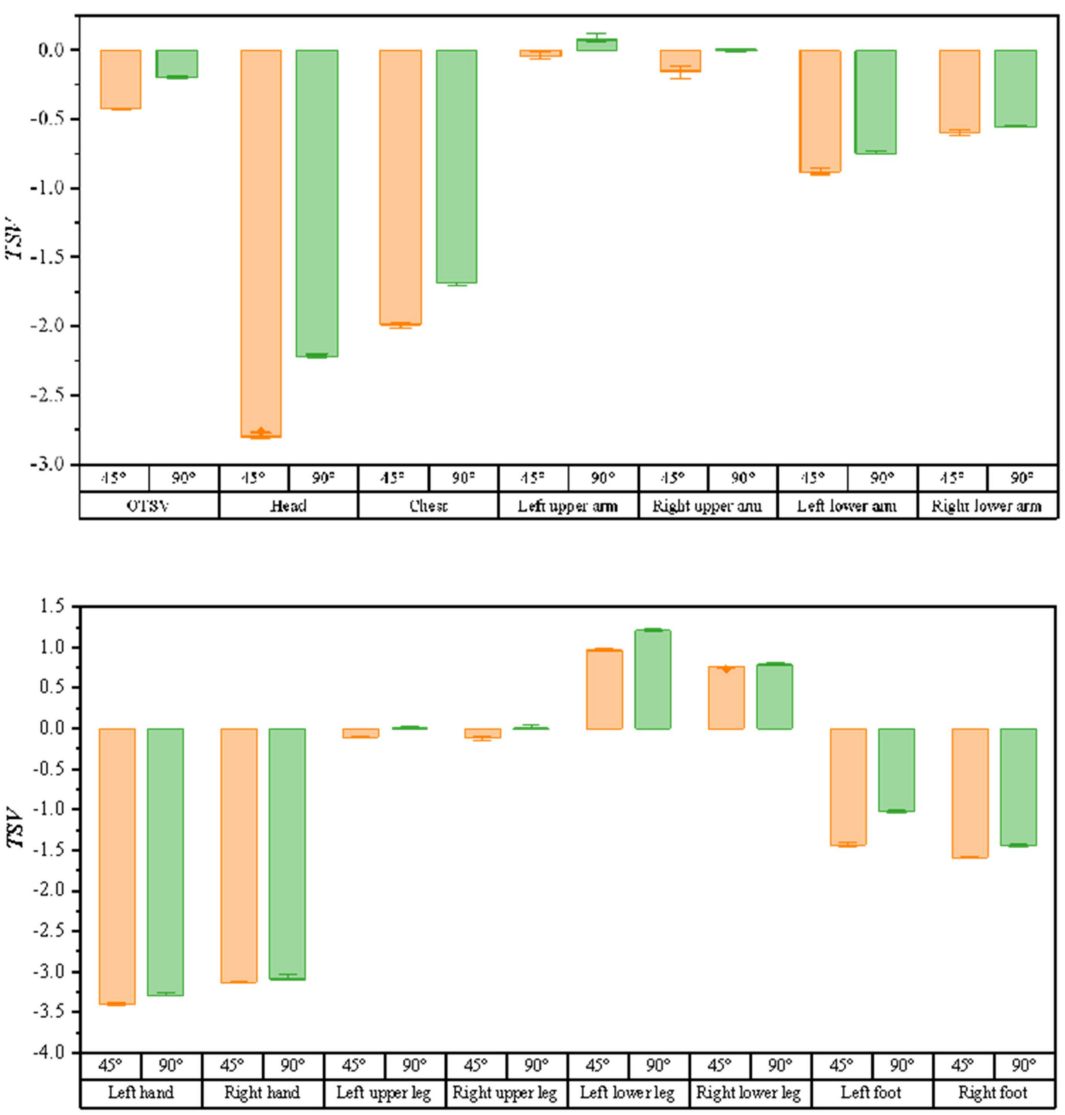
The bar graph of the variation of thermal sensory voting with solar radiation at different air supply angles.

As shown in Figure 8, the bar graphs of thermal sensory voting with solar radiation variation (solar radiation ∈ [900,1,300]) are shown for the air supply angle of 45 degrees and 90 degrees, respectively (when the air supply temperature is 15°, the air supply volume is 600 m^3^/h, and the solar altitude angle is 1 radian). It can be seen from the graph that the TSV at the air supply angle of 90 degrees are larger than the values of the air supply angle of 45 degrees, which means that the driver feels warmer. The correlation between solar radiation and thermal sensory voting was analyzed using Spearman and the results are shown in Table 4. The sites with * indicate that the correlation is not significant at the level of 0.05. From the data in the table, it can be obtained that no correlation with solar radiation and TSV at an air delivery angle of 45°. Comparing the correlation between LTSV and solar radiation for the same body part, the correlation is more significant at an air supply angle of 90°.

**Table 4:** Spearman correlation between solar radiation and TSV at different air supply angles.

45°	OTSV	0.10*	90°	Left hand	0.80
90°	OTSV	0.80	45°	Right hand	0.47
45°	Head	0.03*	90°	Right hand	0.87
90°	Head	−0.21	45°	Left upper leg	−0.34
45°	Chest	−0.62	90°	Left upper leg	1.00
90°	Chest	−0.88	45°	Right upper leg	−0.46
45°	Left upper arm	0.19*	90°	Right upper leg	1.00
90°	Left upper arm	0.93	45°	Left lower leg	0.26
45°	Right upper arm	−0.51	90°	Left lower leg	0.93
90°	Right upper arm	−0.74	45°	Right lower leg	0.87
45°	Left lower arm	0.01*	90°	Right lower leg	0.91
90°	Left lower arm	0.78	45°	Left foot	0.39
45°	Right lower arm	−0.10	90°	Left foot	0.63
90°	Right lower arm	0.97	45°	Right foot	−0.67
45°	Left hand	−0.04*	90°	Right foot	0.90

**Figure 9:**
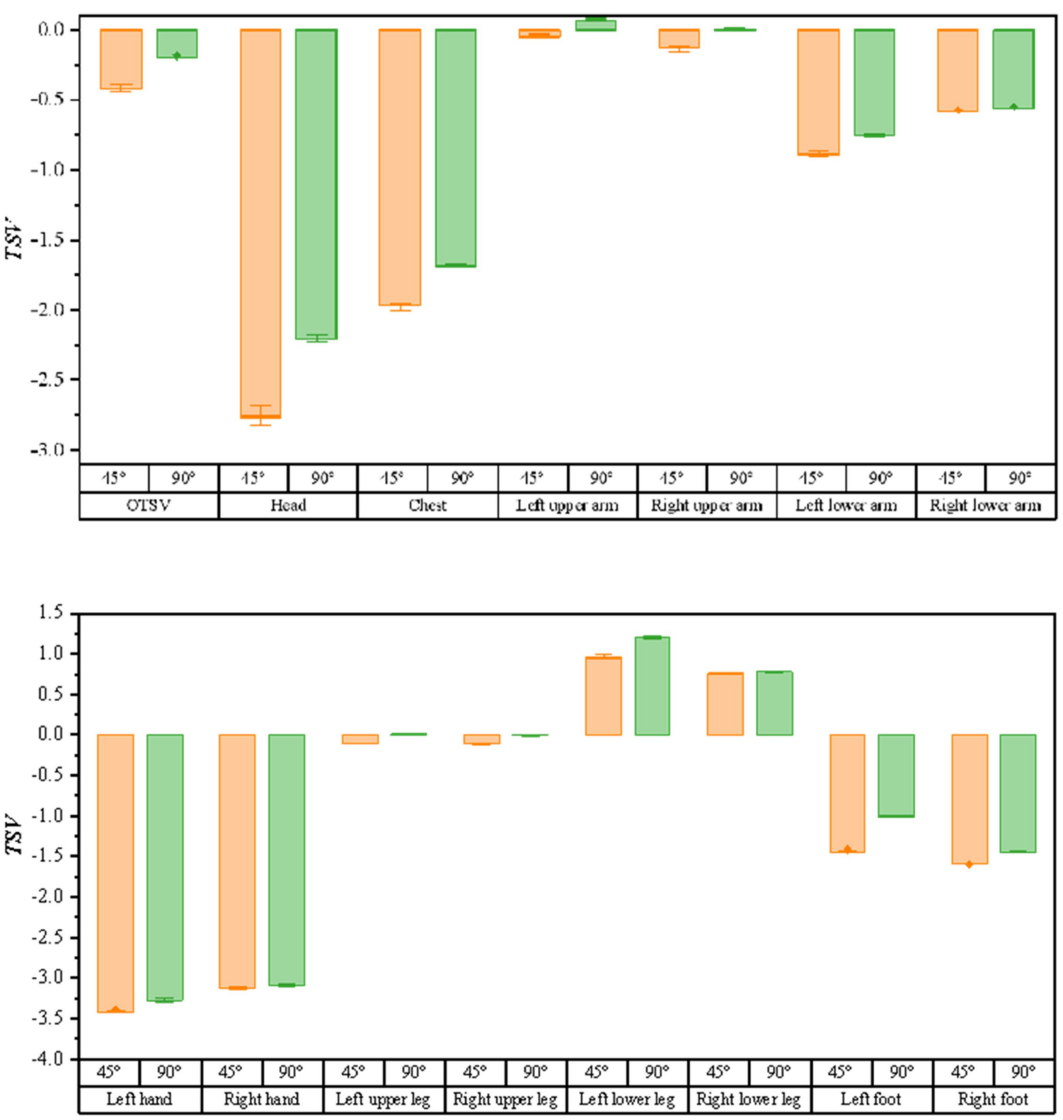
Bar graph of the variation of thermal sensation votes with solar altitude angle for different air supply angles.

As illustrated in Figure 9, the bar graphs of the variation of thermal sensory voting with solar altitude angle (solar altitude angle ∈ [0.8, 1.4]) are shown for the conditions of 45 and 90 degrees of air supply angle (when the air supply temperature is 15°C, the air supply volume is 600 m3/h, and the solar radiation is 1,100 w/m2), respectively. Similarly, as the solar altitude angle varies, the TSV is larger for a supply air angle of 90 degrees than for a supply air angle of 45 degrees. The results of the correlation between the solar altitude angle and thermal sensory voting using Spearman’s analysis are shown in Table 5, where the thermal sensation vote for most body parts had a high correlation with the solar altitude angle.

**Table 5:** Spearman correlation between solar altitude angle and TSV at different air supply angles.

45°	OTSV	0.95	90°	Left hand	0.98
90°	OTSV	−0.72	45°	Right hand	0.78
45°	Head	0.92	90°	Right hand	0.80
90°	Head	0.77	45°	Left upper leg	0.84
45°	Chest	0.98	90°	Left upper leg	0.85
90°	Chest	−0.22	45°	Right upper leg	−0.79
45°	Left upper arm	0.88	90°	Right upper leg	0.78
90°	Left upper arm	0.19*	45°	Left lower leg	0.86
45°	Right upper arm	0.10*	90°	Left lower leg	−0.95
90°	Right upper arm	0.87	45°	Right lower leg	−0.71
45°	Left lower arm	0.87	90°	Right lower leg	−0.73
90°	Left lower arm	0.64	45°	Left foot	0.79
45°	Right lower arm	0.47	90°	Left foot	−0.74
90°	Right lower arm	0.41	45°	Right foot	−0.49
45°	Left hand	−0.68	90°	Right foot	0.12*

### 4.3. Optimization results

**Figure 10:**
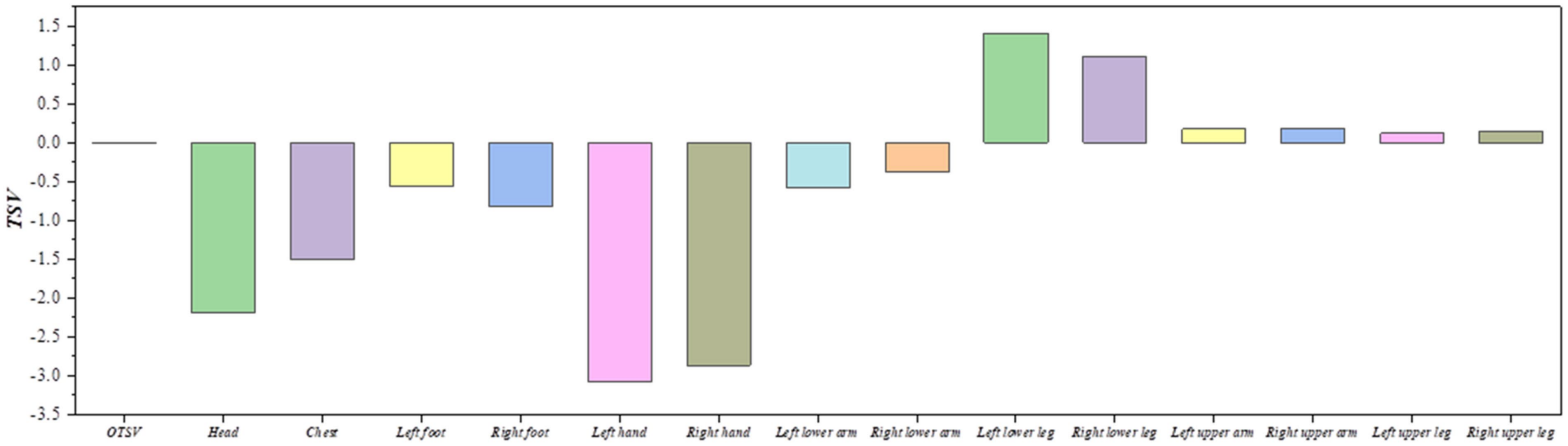
Results of the heat sensing poll with optimal air supply parameters.

In order to have the most comfortable thermal sensation for the train driver, we set the OTSV converges to 0 as the optimization objective. Calculating the optimal results under extreme operating conditions as the solar altitude angle is 1.4 radian and the solar radiation is 1,300 W/m^2^. Constraining the LSTV and OTSV between −4 and + 4. Finally, the optimal air supply parameters were obtained using Isight’s pointer algorithm: air supply angle = 90°, air supply volume = 780 m^3^/h, air supply temperature = 19°C. Figure 10 shows the LTSV and OTSV of each body part under this working condition. The optimized overall thermal sensation is almost neutral (OTSV = 0), which can provide train drivers with a better thermal sensation, improve work efficiency and reduce health and safety risks. In addition, we also used the NLPQLP optimization method for comparison in order to get the best results, and the final results are shown in Table 6. The OTSV was our primary area of concern, therefore it is clear from the results in the table that the pointer algorithm solves for better results.

**Table 6:** The optimal results of Pointer algorithm and NLPQLP algorithm.

	Pointer	NLPQLP
OTSV	0.00	−0.21
Head	−2.19	−2.22
Chest	−1.50	−1.71
Left foot	−0.55	−1.03
Right foot	−0.82	−1.40
Left hand	−3.08	−3.19
Right hand	−2.87	−3.02
Left lower arm	−0.58	−0.74
Right lower arm	−0.38	−0.54
Left lower leg	1.41	1.17
Right lower leg	1.12	0.81
Left upper arm	0.18	0.10
Right upper arm	0.19	0.05
Left upper leg	0.12	0.04
Right upper leg	0.15	0.06

## 5. Conclusion

In this paper, an optimal design scheme for the ventilation system in the train cab was proposed by integrating the pointer optimization algorithm of Isight software with the RBF approximation method. The pointer algorithm enables automatic capture the design space information and the combination of the four optimization algorithms, which simplifies the selection process and saves time. We validated our approach by creating a thermal comfort model of a train cab coupled with the human thermal regulation model, using fine 3D-scanned thermal manikin models to enhance the computational accuracy.

Our analysis reveals that the five input variables significantly affect both the LTSV and the OTSV of the train driver, indicating the inhomogeneity of the thermal environment in the driver’s room. Finally, we obtain the optimal air supply parameters for the HVAC system under extreme summer conditions, which result in a neutral overall thermal sensation for the driver. However, the LTSV of some body parts, such as the head, chest and hands, remains uncomfortable. Thus, achieving synergistic optimization of local and overall thermal sensation is an area for further study.

## Data availability statement

The raw data supporting the conclusions of this article will be made available by the authors, without undue reservation.

## Author contributions

ZY proposed the conceptualization and wrote the methodology with WZ, QX, and XL did the investigation, while ZY and GX carried out the software. ZY and WZ wrote the original manuscript with YP, MY, and CF completed the review and editing. ZY, GX, and XL supervised the project. All authors contributed to the article and approved the submitted version.

## Funding

This study was supported by the “Tenth Five-Year Plan” Major Science and Technology Project of CRRC [Grant number: 2021CKZ008-3].

## Conflict of interest

ZY, GX, and XL are employed by CRRC Industrial Research Institute (Qingdao) Co., Ltd.

The remaining authors declare that the research was conducted in the absence of any commercial or financial relationships that could be construed as a potential conflict of interest.

## Publisher’s note

All claims expressed in this article are solely those of the authors and do not necessarily represent those of their affiliated organizations, or those of the publisher, the editors and the reviewers. Any product that may be evaluated in this article, or claim that may be made by its manufacturer, is not guaranteed or endorsed by the publisher.

## Abbreviations

RBF: Radial basis function
Opt LHD: Optimal Latin Hypercube Design
LTSV: Local thermal sensation vote
OTSV: Overall thermal sensation vote
HVAC: Heating Ventilation and Air Conditioning
OSH: Occupational safety and health
PMV: Predicted mean vote
MST: Mean skin temperature
CFD: Computational Fluid Dynamics
